# Assessment of Zinc Content in Food Supplements

**DOI:** 10.3390/foods15010151

**Published:** 2026-01-02

**Authors:** Anna Puścion-Jakubik, Katarzyna Kolenda, Katarzyna Socha, Renata Markiewicz-Żukowska

**Affiliations:** Department of Bromatology, Faculty of Pharmacy with the Division of Laboratory Medicine, Medical University of Białystok, Mickiewicza 2D Street, 15-222 Białystok, Poland; kolendakate@gmail.com (K.K.); katarzyna.socha@umb.edu.pl (K.S.); renata.markiewicz-zukowska@umb.edu.pl (R.M.-Ż.)

**Keywords:** pharmacy, food supplements, zinc, safety, AAS

## Abstract

Zinc (Zn) is an essential trace element that plays a key role as a cofactor for over 300 enzymes involved in metabolic processes, protein synthesis, and gene expression regulation. Zn supplementation is used in the prevention and treatment of infectious, dermatological, and reproductive system diseases. Legal regulations allow for a relatively wide range of mineral content in this product category (from −20% to +45% of the declared value). The study aimed to analyze the quality of food supplements containing Zn—compliance with declared Zn content was assessed. The study included 80 preparations. The preparations varied in terms of declared Zn content, pharmaceutical form, chemical form of Zn, composition, and primary mode of action. Zn content was determined by atomic absorption spectrometry after prior mineralization of the samples in concentrated nitric acid in a closed microwave system. It was estimated that 70% of food supplements contained Zn within the acceptable range. It should be emphasized that 23.75% of the preparations contained more Zn than the permissible range of Zn content, and 6.25% contained less—both of these groups of preparations may be associated with a health risk. From a regulatory perspective, these results highlight the need for continuous surveillance of the food supplement market to improve consumer safety.

## 1. Introduction

Zinc (Zn) is a mineral essential for the proper functioning of the body. Its role is multifaceted. For example, it contributes to the proper functioning of the immune system in all age groups, including infants and young children. A cause-and-effect relationship has been demonstrated between dietary Zn intake and the proper functioning of the immune system [1,2].

Zn acts as a key structural element in bone tissue, where it participates in the regulation of collagen matrix biosynthesis and mineralization processes, and also plays an important role in dynamic bone remodeling by influencing the activity of osteoblasts and osteoclasts [3].

This mineral plays a crucial role in the proper functioning of the immune system. It participates in cellular and humoral immune responses and acts as an ionic regulator of immune responses. Its deficiency adversely affects the growth and function of T and B lymphocytes, as well as the development of NK cells and neutrophils. Disturbed homeostasis results in increased susceptibility to infections due to impaired immune function [4,5,6,7,8,9]. It has also been observed that chronic Zn deficiency increases the risk of cancer, which allows us to conclude that it plays a role in the defense against the initiation and promotion of cancer [7,10].

An interesting observation is that lower Zn concentrations were found in the serum of people with depression compared to healthy individuals. Zn concentrations correlated with the severity of symptoms [11,12,13,14,15].

Zn absorption is higher from a diet containing animal protein than from a diet containing plant protein [16,17,18].

The Dietary Supplements Team, which assesses the safety of food supplements in Poland, has set the maximum amount of Zn in dietary supplements at 15 mg/day for an adult [18].

The latest Zn intake standards for the Polish population at the EAR level are: 2.5 mg (for infants aged 6–11 months and 1–3 years), 4 mg (children aged 4–6 years and 7–9 years), 6.8 mg (women from 19 years of age), 7 mg (boys and girls aged 10–12 years), 7.3 mg (girls aged 13–15 mg and 16–18 years), 8.5 mg (boys 13–18 years), 9.4 mg (men from 19 years of age), 9.5 mg (pregnant women aged 19 years and over), 10.4 mg (breastfeeding women aged 19 years and over), 10.5 mg (pregnant women under 19 years of age), 10.9 mg (breastfeeding women under 19 years of age) [17].

The standards published by EFSA take into account the share of phytates in the diet. At the request of the European Commission, the Panel on Dietetic Products, Nutrition and Allergies (NDA) established reference intakes for Zn, adopting a two-step factorial approach and reference values based on body weight. The average requirements are 6.2–10.2 mg/day for women with a reference weight of 58.5 kg and 7.5–12.7 mg/day for men with a reference weight of 68.1 kg. During pregnancy and lactation, additional Zn requirements related to fetal and maternal tissues and the excretion of this mineral in breast milk are taken into account [2].

Health problems resulting from Zn deficiency were described as early as 1961. Anemia, hypogonadism, and dwarfism were observed in young men living in Iran and Egypt. The clinical picture was linked to their diet, based primarily on bread, beans, potatoes, and milk. Introducing protein-rich foods into the diet and administering Zn supplements corrected the hypogonadism and dwarfism. This case was the starting point for a growing interest in the clinical aspects of Zn use [8].

Therefore, the aim of this study was to analytically determine the Zn content in food supplements. The next stage of this project was the evaluation of the content in the context of compliance with manufacturers’ declarations and recommendations, which enabled verification of the quality (compliance with declared Zn content) and safety of use of the preparations. This issue is very important from the point of view of public health, especially in the context of the safety of supplementation and the possible impact on patients’ pharmacotherapy.

## 2. Materials and Methods

### 2.1. Materials

In order to assess the quality (compliance with declared Zn content) of food supplements containing Zn, 80 preparations available in Poland were selected and purchased in stationary and online pharmacies. The following inclusion criteria were adopted: popularity of the preparations in online pharmacy rankings, diversity of the pharmaceutical form of the preparations and diversity of the chemical form of Zn. For each preparation, analyses were performed three times.

### 2.2. Sample Homogenization and Mineralization

Samples of food supplements were homogenized (in a vibratory mill, Testchem, Radlin, Poland) to obtain a homogeneous mass. Then, approximately 0.3 g of the mass was weighed on a precision scale, with an accuracy of 1 mg, into teflon mineralization vessels. After adding 4 mL of spectrally pure concentrated 69% nitric acid (Tracepur, Merck, Darmstadt, Germany), the microwave mineralization process was carried out in a closed system (mineralizer Berghof, Speedwave, Eningen, Germany). Mineralization proceeded according to the scheme (time/pressure/temperature/% of microwave power):

- step 1: 10 min/20 atm./170 °C/80%;
- step 2: 10 min/30 atm./190 °C/90%;
- step 3: 10 min/40 atm./210 °C/90%;
- step 4: 18 min/40 atm./50 °C/0%.

Mineralizates were quantitatively transferred to polypropylene vessels using deionized water prepared with Simplicity 185 (Millipore, Burlington, VT, USA), and appropriate dilutions were prepared in accordance with the range of the standard curve—the dilution of each sample was selected individually based on the manufacturer’s declaration of Zn content.

### 2.3. Determination of Zn Content and Validation of AAS Method

The Zn content was determined using the AAS (atomic absorption spectrometry) method. They were carried out using a Z-2000 spectrophotometer (Hitachi, Tokyo, Japan) with Zeeman background correction. The absorption measurement was made at a wavelength of 285.2 nm. To transform the sample into the state of free atoms, atomization in an acetylene-air flame was used. Working solution calibration curves were made from a standard solution with a concentration of 1 g/L (Merck, Darmstadt, Germany). Each marking was repeated three times.

To validate the method, a certified reference material was used (Mixed Polish Herbs NCT-MPH-2, LIVSMEDELS VERKET, National Food Administration, Uppsala, Sweden). All obtained Zn values (determined before sample analysis and every 10 samples) were within the certified range (from 31.4 to 35.6 mg/kg). The coefficient of variation was V = 4.70% and the accuracy (% error): 1.6. LOD (limit of detection) was 0.018 mg/kg, LOQ (limit of quantification) was 0.054 mg/kg [19,20].

### 2.4. Data Analysis

The computer program Statistica v. 13.3 (Tibco Software Inc., Palo-Alto, CA, USA) and Excel (Microsoft Office) were used to prepare and statistically analyze the obtained results. The values of arithmetic means (Av.) with standard deviations (SD), minimum (Min) and maximum (Max) values, medians (Med.), lower (Q1) and upper quartiles (Q3) were determined. The normality of the distribution of the obtained numerical results was assessed using the Shapiro–Wilk test. The chi-square test was used to assess differences between groups of samples. Differences at the significance level of *p* < 0.05 were considered statistically significant results.

Moreover, the obtained results regarding the Zn content in a serving were referred to the standards, which indicate that the permissible range of mineral content in food supplements is between −20% and +45% of the content declared by the manufacturer [19].

## 3. Results

### 3.1. Zn Content in One Serving

The results regarding the Zn content in food supplements, taking into account the adopted division criteria, are presented in Table 1. The following criteria were used to conduct the analyses: declared Zn content, pharmaceutical and chemical form, composition and main purpose of using supplements with this mineral.

Considering the declared Zn content, it was shown that in the group of preparations with a Zn content of less than 5 mg, the highest measured content was 9.7 mg/serving, and Q3 was 5.0 mg/serving.

In the group of preparations with a content of up to 15 mg, the maximum value was 22.9 mg/serving, and Q3 was 12.6 mg/serving.

Another criterion was the pharmaceutical form. Tablets (*n* = 35) were the most common form, with a Zn content ranging from 1.3/serving to 19.6 mg/serving. Capsules (*n* = 19) were the second most popular form, with a Zn content ranging from 2.7/serving to 28.6 mg/serving.

Zn oxide was the most common form of Zn in food supplements (*n* = 26), with contents ranging from 1.3/serving to 22.9 mg/serving. Other common chemical forms include Zn gluconate (*n* = 17), Zn sulfate (*n* = 13), and Zn citrate (*n* = 12).

The vast majority of the supplements analyzed were multi-ingredient preparations (*n* = 70). Their content of the tested mineral ranged between 1.2 mg/serving and 24.2 mg/serving.

The most popular uses of Zn food supplements include their effect on memory and concentration (*n* = 14) and on hair, skin, and nails (*n* = 14). The maximum values in these supplement groups were 15.4 mg/serving and 19.1 mg/serving.

### 3.2. Relating the Obtained Results to the Norms

Analysis of Zn content in one serving of food supplement showed that 70% of preparations were within the acceptable range (i.e., from −20 to +45% of the declared value). Of the preparations tested, 6.25% contained Zn below the acceptable range, and 23.75% above it. A statistically significant correlation was found between the pharmaceutical form of food supplements and the compliance of Zn content with the manufacturer’s declaration. No such correlation was observed for the other criteria used (declared content, chemical form, amount of minerals in the preparation, and the main category of the preparation’s intended use) (Table 2).

### 3.3. Safety Assessment of Food Supplements Containing Zinc

**Figure 1:**
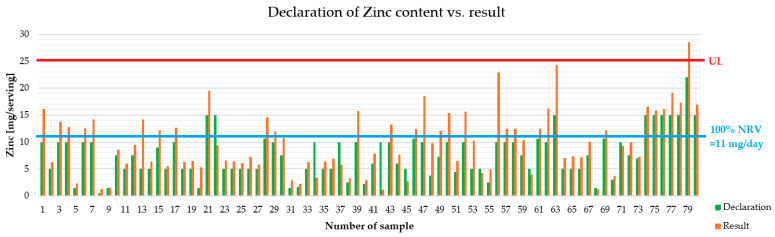
Comparison of declared and marked Zn contents for all tested food supplements. NRV—Nutrient Reference Value.

Figure 1 presents the declared values in the serving and the labeled values for each dietary supplement. Furthermore, the UL (upper limit) and average requirement (100% NRV = 11 mg per day) are indicated. In Poland, the Dietary Supplements Committee has established that the maximum allowable Zn content in food supplements is 15 mg.

**Figure 2:**
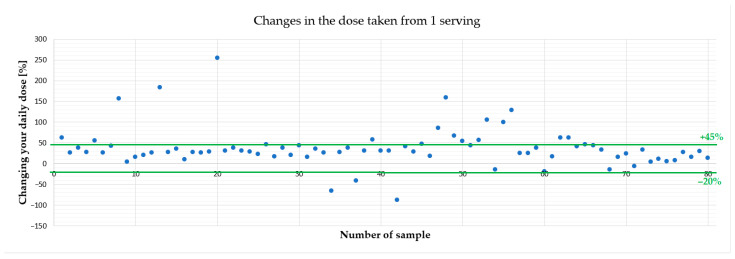
Discrepancy between the declared values of Zn content and the results (permissible deviations are marked with green lines).

Figure 2 presents deviations for individual food supplements - we have shown that preparations containing even more than 250% of the declared Zn content were available on the market.

## 4. Discussion

The quality of food supplements—especially mineral supplements containing ingredients like Zn—is a key but often underestimated factor in both efficacy and safety of therapy. Zn is a trace element involved in numerous enzymatic reactions, immune system function, and antioxidant defenses. Therefore, adequate intake, consistent with recommendations for specific groups, is crucial for health [1,2]. At the same time, regulations allow for relatively wide tolerances between labeled and actual mineral content, meaning that food supplements may sometimes contain significantly less—or more—Zn than the declared amount.

In case of dietary deficiencies, patients’ first choice should be to reach for products rich in zinc. Examples of foods rich in Zn include: oysters, meat, especially liver, nuts (e.g., cashews, almonds), pumpkin seeds, cocoa and whole grain products [16]. Zn absorption from the diet is 20–40%, and increases in the presence of Zn deficiency. Zn absorption is enhanced by certain amino acids and citric acid, while it is reduced by the presence of phytates, oxalates, and fiber, as well as other minerals such as calcium and copper [17].

Literature data indicate that Zn performs regulatory and catalytic functions in the human body. It is a component of over 300 enzymes involved in the biosynthesis of various components, including proteins. It also participates in the metabolism of proteins, carbohydrates, and fats. It is responsible for maintaining the stability of cell membranes. Furthermore, it is responsible for the sense of taste and smell. It also participates in learning and memory processes, influencing, among other things, synaptic plasticity [18].

If it is not possible to supplement deficiencies through diet, patients can choose food supplements—preferably those available in pharmacies. According to COMMISSION REGULATION (EC) No 1170/2009 of 30 November 2009, Zn may be present in food supplements in the following chemical forms: zinc acetate, zinc L-ascorbate, zinc L-aspartate, zinc bisglycinate, zinc chloride, zinc citrate, zinc gluconate, zinc lactate, zinc L-lysinate, zinc malate, zinc mono-L-methionine sulfate, zinc oxide, zinc carbonate, zinc L-pidolate, zinc picolinate, zinc sulfate [21].

The demand for Zn is determined by a number of biological and dietary factors, including the degree of bioavailability of this element from food, its antagonistic and synergistic interactions with other micronutrients, the size of the endogenous Zn pool accumulated in the body, and the amount of losses resulting from excretory processes, such as excretion in feces, urine, or sweat [17].

In this study, the Zn content in food supplements was compared with the value stated on the label, using a tolerance range of −20% to +45% of the declared mineral content in food supplements [21]. In this context, 70% of the products analyzed were within acceptable limits, 6.25% contained less Zn than permitted (below −20% of the declared value), and 23.75% contained more than the permitted range (above +45% of the declared value), as presented in Table 2 and Figure 2. These results are broadly consistent with other European data suggesting that most products are within acceptable limits, but a small minority significantly deviate from label claims. For example, a study conducted by the Food Safety Authority of Ireland (FSAI) found that 86% of the food supplements tested were within acceptable limits, but one product containing Zn exceeded the tolerance limit (+45%) and three preparations contained amounts below the lower limit (−20%) [22].

From a regulatory perspective, a wide tolerance range is intended to account for analytical variability, manufacturing variations, and, for example, degradation of ingredients during shelf life. However, the relatively wide range of acceptable values for minerals means that even a product that complies with current standards can deviate significantly from its declared value. For a food supplement with a declared Zn content of 15 mg, the acceptable range is 12 to 22 mg. Chronic daily use of a supplement containing Zn at levels near the upper end of this range, especially in combination with Zn from diet and other supplements, may approach or exceed the Tolerable Upper Intake Limit (UL) for adults, which was set at 25 mg per day in the EU by the previous Scientific Committee on Food and approved by EFSA. Therefore, even if most products meet formal tolerance criteria, they may still pose a potential safety risk in realistic use scenarios [1,2]. It is important to note that one preparation exceeded the UL—this is important information for geriatric patients, for example, who may be affected by multi-medication and multi-morbidity. Preparations intended for children should also be subject to special scrutiny due to the sensitivity of this group.

Of all food supplements analyzed in this study, 6.25% of the preparations had insufficient Zn content, raising concerns primarily about the effectiveness of supplementation. If a product provides significantly less Zn than advertised, individuals using supplementation to correct significant deficiencies or meet increased requirements (e.g., vegetarians, individuals with malabsorption, or certain patient groups) may not achieve the appropriate clinical effect. Clinical consequences of chronic Zn deficiency include impaired immune response, delayed wound healing, impaired taste and smell, and impaired growth and reproductive function [23].

More problematic from a safety perspective is the fact that as many as 23.75% of the products contained Zn in amounts exceeding the upper tolerance limit of +45% of the declared value. For a nominal dose of 15 mg of Zn, this translates to an actual Zn content above approximately 22 mg per daily dose; for products that already declare relatively high doses (e.g., 20–25 mg), such excesses may result in actual intake approaching or exceeding the UL, particularly in individuals consuming a Zn-rich diet or taking many products containing Zn (e.g., “immune-boosting” preparations) [24].

Chronic excessive Zn intake is associated with several potential negative health consequences—this aspect was not investigated in this project, but there are many publications pointing to this problem. High Zn intake may interfere with copper absorption by inducing intestinal metallothionein, which preferentially binds copper and reduces its systemic availability [24]. Copper deficiency, in turn, can lead to anemia, neutropenia, myelopathy, and sensory neuropathy, and is associated with changes in lipid metabolism and hemostasis markers. However, it should be emphasized that this deficiency is not common in the general population. Some studies suggest that a high Zn/Cu ratio is associated with lower HDL cholesterol levels and an increased incidence of metabolic syndrome, further raising concerns about long-term high-dose Zn supplementation in populations already at cardiometabolic risk [25].

Beyond the issue of total Zn content, chemical forms and their bioavailability should also be considered. Different salts (e.g., Zn gluconate, sulfate, oxide, and citrate) and organic complexes differ in their solubility and behavior in the gastrointestinal tract, and in vitro studies show significant differences in Zn bioavailability from different supplement formulations and matrices. A product that meets the declared total Zn content may nevertheless have low bioavailability, reducing its clinical utility. Conversely, highly bioavailable forms in high-content preparations increase the risk of exceeding physiological requirements. Therefore, the quality assessment of Zn supplements should consider both the composition and bioavailability assessment, the declared chemical form, and the dosage in relation to established reference intakes [26,27].

The results of this study are consistent with those of other studies assessing the quality of mineral supplements. Analyses of Zn content in popular food supplements available in Poland revealed discrepancies between labeled and declared values, although the magnitude and direction of variations vary depending on the product and manufacturer. For example, one study assessed the quality of 42 multi-ingredient food supplements. The declared Zn content ranged from 0.5 to 15.6 mg per tablet. The authors indicated that the Zn content in all the food supplements tested was lower than the manufacturer’s declared value [28]. The advantage of our study is that it encompasses a wider range of commercially available supplements.

Another publication assessed the quality of food supplements intended for athletes in terms of their Zn content, among other things. The study included six preparations, of which one food supplement had a declared Zn content 132.5% higher than the recommended daily intake [29]. These data highlight the importance of quality control systems, validated analytical methods and routine post-market surveillance of food supplements.

Variations between declared and actual Zn content in food supplements may result from numerous concurrent factors, including technological, organizational, and regulatory aspects. In practice, manufacturers may use so-called safety margins (overages) to maintain the declared Zn content throughout the product’s shelf life, but excessive use can lead to unintentional exceedance of recommended intake levels. On the other hand, underestimated Zn content may be a consequence of insufficient quality control, ingredient degradation during storage, or efforts to reduce production costs. Furthermore, the current model of food supplement supervision, based primarily on post-registration control, promotes quality variability between batches and manufacturers.

From a public health perspective, the observed problem—most products being within tolerance, but a small number of preparations with levels below the declared value and some with levels higher than the acceptable range—has several implications. For example, physicians and pharmacists cannot assume that the labeled levels accurately reflect the dose taken, which may be a more widespread problem with products purchased online. Furthermore, patient education should emphasize that “more is not better” when it comes to supplementation, and long-term use of high-dose Zn supplements, especially in combination with other products, should be under medical supervision.

From a regulatory perspective, these results highlight the importance of continuous market surveillance, post-marketing controls, and harmonized quality requirements to ensure compliance with declared contents and consumer safety. For manufacturers, these results underscore the need for rigorous quality control and improved process standardization to maintain product consistency and consumer confidence. From a clinical perspective, healthcare professionals should be aware of potential discrepancies between labeled Zn content and actual Zn content when providing advice to patients, especially those with chronic diseases or at risk of micronutrient deficiencies. Finally, from a consumer perspective, increased awareness and health literacy are essential to promote informed decision-making and responsible use of food supplements.

Our analysis of Zn content in food supplements has certain limitations. The study covered a selected group of food supplements available on the market at a given time, which may not fully reflect the total variability of products available on the market, especially with regard to new batches; however, it indicates market trends. The number of food supplements analyzed in each category (e.g., in the category covering pharmaceutical forms) was limited, which could have affected the statistical significance and the ability to detect differences between groups. The effect of storage conditions (temperature, humidity, exposure to sunlight, the presence of various minerals) on the stability of Zn content was not analyzed, which could be important for some products. Furthermore, it should be emphasized that in our project, we determined the total Zn content, not its bioavailability, which may significantly differ depending on the chemical form used (e.g., bioavailability from chelates is better than from oxides), the presence of excipients or interactions with other ingredients of the preparation. Total Zn content is not the only factor determining biological effects.

In summary, this study confirms that although most Zn supplements meet current European tolerance criteria, some significantly deviate from their declared levels, with both under- and over-consumption observed. Products with too low a dose may not prevent or correct Zn deficiency, while those with too high a content of Zn, especially when combined with other dietary sources or when multiple preparations are used simultaneously, may increase the risk of exceeding the UL and causing copper deficiency, lipid profile disturbances, and other adverse effects. Improving the quality of Zn supplements therefore requires rigorous production control, independent analytical verification, and transparent risk communication to healthcare professionals and patients. Future studies should continue to monitor commercially available products. Furthermore, they should focus on assessing the consequences of long-term exposure to high levels of Zn and include analyses of the relationship between Zn dose, bioavailability, and interactions with other minerals such as copper and iron.

## 5. Conclusions

The analyses conducted indicate that, in a subset of the evaluated food supplements, discrepancies were observed between the declared Zn content and the values determined using the applied analytical method. Although most supplements met tolerance criteria, the presence of both under- and over-declared Zn levels highlights the need for continued attention to product quality. Given that consumers may use more than one Zn-containing food supplement simultaneously, these findings underscore the importance of appropriate market oversight and informed use of supplementation.

Future research should focus on assessments of Zn content across different production batches, as well as on the evaluation of Zn bioavailability and chemical forms present in food supplements. Additionally, strengthening post-marketing surveillance systems may contribute to improved compliance with labeling requirements and enhanced consumer safety. From a practical perspective, pharmacists and other healthcare professionals play a key role in advising patients on the safe and rational use of Zn-containing supplements, particularly in the context of long-term use and potential cumulative exposure.

## Figures and Tables

**Table 1:** Determined Zn content (mg) per serving in food supplements depending on various criteria (serving = 1 tablet, capsule or other single form enabling dosing).

	*n*	Zn Content (mg/Serving)			
		Av. ± SD	Min–Max	Med.	Q1–Q3
Declared content					
≤5 mg	13	3.7 ± 2.4	1.3–9.7	2.9	2.3–5.0
5 mg–15 mg	57	9.8 ± 4.3	1.2–22.9	9.5	6.4–12.6
≥15 mg	10	18.4 ± 5.1	9.4–28.6	17.1	16.1–19.6
Pharmaceutical Form					
Capsules	19	12.5 ± 6.9	2.7–28.6	12.5	5.9–16.2
Tablets	35	8.8 ± 5.3	1.3–19.6	6.9	4.3–13.7
Effervescent tablets	11	8.2 ± 3.9	1.6–12.6	10.0	5.5–12.0
Coated tablets	9	8.7 ± 3.9	1.2–13.2	9.4	6.4–12.5
Lozenges	3	10.5 ± 5.8	7.1–17.3	7.3	7.0–17.3
Other	3	14.5 ± 9.0	6.4–24.2	12.7	6.4–24.2
Chemical form					
Zn oxide	26	8.2 ± 5.2	1.3–22.9	6.4	5.9–10.3
Zn gluconate	17	11.4 ± 5.3	2.9–19.1	12.1	7.1–16.1
Zn sulfate	13	6.7 ± 4.8	1.2–14.6	5.0	2.9–12.2
Zn citrate	12	11.1 ± 5.4	5.5–24.2	10.0	7.1–13.0
Zn bisglycinate	9	13.5 ± 3.6	6.4–17.3	14.2	12.7–15.8
Other	3	14.1 ± 12.8	4.0–28.6	9.7	6.9–19.1
Composition					
Only Zn	10	15.7 ± 6.0	7.3–28.6	16.4	11.4–17.2
Multimineral preparations	70	9.1 ± 5.2	1.2–24.2	7.5	5.8–12.5
Purpose of the supplementation					
Increasing physical fitness	9	9.6 ± 6.4	2.7–22.9	7.3	6.4–12.6
Improving memory and concentration	14	8.7 ± 4.5	1.2–15.4	7.4	6.1–12.3
Supporting the treatment of osteoporosis	4	11.6 ± 4.4	6.4–16.1	11.8	8.7–14.7
Affecting the condition of hair, skin and nails	14	8.3 ± 5.9	1.3–19.1	6.2	4.5–11.0
Increasing immunity	8	13.1 ± 7.3	5.8–28.6	11.2	8.7–15.3
For women	6	10.8 ± 4.8	3.4–15.8	12.7	7.9–13.5
No specific purpose	25	9.9 ± 5.9	1.3–24.2	10.0	6.3–12.7
TOTAL	80	9.90 ± 5.71	1.15–28.55	9.37	6.06–13.49

Av.—average, Max—maximum value, Med.—median, Min—minimum value, Q1—lower quartile, Q3—upper quartile, SD—standard deviation.

**Table 2:** Percentage of food supplements with the Zn content below and above the declared value and within the permissible range, taking into account selected criteria (chi-square test).

Criterion	Subgroup	*n* (Total)	Below Norm *n* (%)	Permissible Range *n* (%)	Above Normal *n* (%)
Declared content	≤5 mg	13	0 (0.00)	7 (8.75)	6 (7.50)
	5 mg–15 mg	57	4 (5.00)	41 (51.25)	12 (15.00)
	≥15 mg	10	1 (1.25)	8 (10.00)	1 (1.25)
Pharmaceutical Form *	Capsules	19	2 (2.50)	6 (7.50)	11 (13.75)
	Tablets	35	1 (1.25)	28 (35.00)	6 (7.50)
	Effervescent tablets	11	0 (0.00)	10 (12.50)	1 (1.25)
	Coated tablets	9	2 (2.50)	7 (8.75)	0 (0.00)
	Lozenges	3	0 (0.00)	3 (3.75)	0 (0.00)
	Other	3	0 (0.00)	2 (2.50)	1 (1.25)
Chemical form	Zn oxide	26	1 (1.25)	20 (25.00)	5 (6.25)
	Zn gluconate	17	0 (0.00)	13 (16.25)	4 (5.00)
	Zn sulfate	13	2 (2.50)	8 (10.00)	3 (3.75)
	Zn citrate	12	2 (2.50)	7 (8.75)	3 (3.75)
	Zn bisglycinate	9	0 (0.00)	6 (7.50)	3 (3.75)
	Other	3	0 (0.00)	2 (2.50)	1 (1.25)
Composition	Only Zn	10	0 (0.00)	10 (12.50)	0 (0.00)
	Multimineral preparations	70	5 (6.25)	46 (57.50)	19 (23.75)
Purpose of the supplementation	Increasing physical fitness	9	2 (2.50)	4 (5.00)	3 (3.75)
	Improving memory and concentration	14	0 (0.00)	10 (12.50)	4 (5.00)
	Supporting the treatment of osteoporosis	4	0 (0.00)	3 (3.75)	1 (1.25)
	Affecting the condition of hair, skin and nails	14	1 (1.25)	8 (10.00)	5 (6.25)
	Increasing immunity	8	0 (0.00)	8 (10.00)	0 (0.00)
	For women	6	0 (0.00)	5 (6.25)	1 (1.25)
	No specific purpose	25	2 (2.50)	18 (22.50)	5 (6.25)
TOTAL			5 (6.25)	56 (70.00)	19 (23.75)

* *p* < 0.01

## Data Availability

The original contributions presented in the study are included in the article, further inquiries can be directed to the corresponding author.

## Abbreviations

The following abbreviations are used in this manuscript:

UL	Tolerable Upper Intake Level
Zn	Zinc
